# Theories and Methods for Labeling Cognitive Workload: Classification and Transfer Learning

**DOI:** 10.3389/fnhum.2019.00295

**Published:** 2019-09-11

**Authors:** Ryan McKendrick, Bradley Feest, Amanda Harwood, Brian Falcone

**Affiliations:** Human-Machine Teaming, Advanced Intelligent Systems, Mission Systems, Northrop Grumman Corporation, McLean, VA, United States

**Keywords:** mental workload, transfer learning, brain–computer interface, functional near-infrared spectroscopy, prefrontal cortex

## Abstract

There are a number of key data-centric questions that must be answered when developing classifiers for operator functional states. “Should a supervised or unsupervised learning approach be used? What degree of labeling and transformation must be performed on the data? What are the trade-offs between algorithm flexibility and model interpretability, as generally these features are at odds?” Here, we focus exclusively on the labeling of cognitive load data for supervised learning. We explored three methods of labeling cognitive states for three-state classification. The first method labels states derived from a tertiary split of trial difficulty during a spatial memory task. The second method was more adaptive; it employed a mixed-effects stress–strain curve and estimated an individual’s performance asymptotes with respect to the same spatial memory task. The final method was similar to the second approach; however, it employed a mixed-effects Rasch model to estimate individual capacity limits within the context of item response theory for the spatial memory task. To assess the strength of each of these labeling approaches, we compared the area under the curve (AUC) for receiver operating curves (ROCs) from elastic net and random forest classifiers. We chose these classifiers based on a combination of interpretability, flexibility, and past modeling success. We tested these techniques across two groups of individuals and two tasks to test the effects of different labeling techniques on cross-person and cross-task transfer. Overall, we observed that the Rasch model labeling paired with a random forest classifier led to the best model fits and showed evidence of both cross-person and cross-task transfer.

## Introduction

People can have “off days” where even the simplest tasks seem difficult, or days where they are “in the zone” and tasks that would normally take hours are quick and easy. Being “off” or “in the zone” are poorly defined common terms used to express a person’s current state of mind. We are able to use these vague terms to express our state of mind to each other. However, as automation and advanced intelligent systems become commonplace, there is a growing need to be able to precisely communicate a person’s state of mind to these systems. A particularly interesting construct of state of mind is mental workload. The field of human factors commonly discusses three mental workload states: mental overload/task saturation, mental underload, and adequate load. There is contention regarding the correct scientific definition of mental workload; however, there is a degree of acceptance around the following: mental workload is a product of the demand/s of the task and the capacity/ies of the person performing the task, where demands and capacities may be moderated by context (Young et al., 2015). Using this definition of mental workload, we have tested several methods of labeling mental workload and building models to classify a person’s mental workload in real-time. Below, we discuss mental workload states, methods for measuring mental workload, and a brief overview of the various techniques we compared for data labeling, data balancing, machine learning algorithms, and our optimization parameters.

Mental workload in humans can be seen as analogous to the stress–strain relationships seen in physical materials. This may seem odd at first, but physical materials have a limit to the stress they can endure without failing which interacts with the environment. For example, the behavior of a beam that is attached at one end but free on the other is a function of its weight-bearing capacity, the mass placed on its free end (i.e., task demands), and environmental factors such as temperature. Materials are rated for a maximum load with an exact cutoff. A successful construction company uses exactly the correct material for the job. If they use more durable—and expensive—materials than the job requires, they reduce their profit margins. If they use a material too weak for the load, the material will fail. Similarly, we want to utilize human capital as efficiently and effectively as possible. Ideally, all employees would be adequately mentally loaded—given the correct amount and type of work—at all times. Adequate load, unlike mental overload, is obviously a positive state, one where we can accomplish what we set out to do. Under the best conditions, it can also be accompanied by interesting sensations such as euphoria, focus, and purpose (Csikszentmihalyi and Csikszentmihalyi, 1992). However, some employees receive too difficult or too much work, leaving them “overloaded,” while others receive too little or too easy work and are “underloaded.”

The ability to measure and identify mental workload states has utility for maximizing safety, efficiency, performance, and perhaps even well-being. It is well documented that mental overload is an adverse state leading to slow and poor performance. Loss of life in the fields of air and ground transportation is often attributed to mental overload or task saturation (Sumalt et al., 2019). Retrospectively, we all know the feeling of being overloaded, not knowing what to do next, feeling frozen, mentally cloudy, upside-down, or being buried under the task at hand.

Mental underload is an elusive and adverse state. It has been argued that mental underload is more difficult to observe than mental overload, but its effects on performance are no less potent, and given that it often occurs unnoticed makes it even more devastating (Hancock et al., 1995). Colloquially, we might liken mental underload to boredom. It’s the feeling in the back of our mind when driving on a lonely country road, feeling the compulsion to look away from the road, perhaps at some houses or someone doing yard work. This occurs even though we know we should be focused on what is on the road in front of us. This is what is meant when mental underload is referenced as inducing inefficient attentional strategies (Young and Stanton, 2002). Under these effects, it only takes one moment of misused attention in the wrong setting for a catastrophe to occur. Thus, accurately measuring and classifying mental workload is vital to safety.

### Measuring Mental Workload in Humans

There are many methods for measuring mental workload in humans, but they, generally, fall into one of three categories: (1) self-report, (2) behavioral secondary tasks, or (3) physiological measurement. The neuroergonomic approach to mental capacity measurement stresses the integration of measurement types from cognitive neuroscience, cognitive psychology, and human factors to study the brain in relation to performance at work and in everyday settings (Parasuraman and Rizzo, 2008). Essentially, taking the best of subjective, behavioral, and physiological measurement in an appropriate context to synthesize a holistic view of a mental capacity. Neuroergonomics provides the needed tools for non-invasive quantification of relevant cognitive concepts and translating those into design recommendations.

Self-report measures or subjective evaluation is practiced more regularly than other methods primarily due to the ease and cost of administration. The most used subjective tool is the National Aeronautics and Space Association Task Load Index (NASA TLX). The NASA TLX is so widely used that the tool has become synonymous with the concept of mental workload (de Winter, 2014). However, the success of the NASA TLX has not been without criticism. These include how it integrates sources of load (Byers, 1989), its sensitivity to changes in workload over time (Thornton, 1985; Miyake, 1997), how the NASA TLX itself can be a source of workload (Monfort et al., 2018), and the tool lacking construct validity (McKendrick and Cherry, 2018). It has also been argued that there is no external validation for what subjective workload tools measure (Hart and Staveland, 1988). Additionally, self-report measures must be administered retrospectively, even if during a brief pause in the task, and are subject to lapses in memory and recency bias. Due to these shortcomings, subjective metrics are often combined with behavioral measurements.

Secondary task performance is the most common means of behavioral assessment of mental workload. The theory being that the decrement seen in performance on the secondary task is due, primarily, to the combined task load exceeding an individual’s mental workload capacity. The magnitude of this decrement is taken to represent the workload required of the primary task (Gopher, 1993; Wickens, 2008). However, dual-task decrements have been criticized as performance varies with resource allocation, but resources are only inferred from performance (Navon, 1984). Thus, a further advancement in mental workload measurement is related to measurement of the brain’s processing of resources.

Neuroergonomics uses non-invasive neurophysiological tools to measure known correlates of mental effort to assess workload *during a task.* In order to do work, the brain requires oxygen and glucose, which is supplied through the bloodstream; thus, when a brain region works harder, it uses more oxygen and glucose and has increased blood flow. We can monitor this process through non-invasive methods; here, we use functional near-infrared spectroscopy (fNIRS) as it measures the changes in oxygenated and deoxygenated hemoglobin by functional brain regions or, more simply, changes in brain activity. Specifically, we use fNIRS over the prefrontal cortex (PFC), which has a functional relationship with working memory (WM) (Braver et al., 1997; Cohen et al., 1997), decision making (Ramnani and Owen, 2004; Figner et al., 2010), and executive control (Badre et al., 2005; Badre and Wagner, 2007). Tasks that manipulate workload by increasing the number of stimuli or complexity of a task have reported a positive relationship with PFC activity (Ayaz et al., 2011; Bogler et al., 2014; Derosière et al., 2014; Herff et al., 2014). Interestingly, experiments where overload is induced have produced a negative quadratic or ‘inverted u” relationship with PFC activity (Durantin et al., 2014; McKendrick et al., 2014). The effective measurement of mental workload via brain metabolism using fNIRS has been successful in both laboratory settings and complex natural settings (James et al., 2011; McKendrick et al., 2016, 2017).

Machine learning has been used to successfully classify mental workload states *via* brain activity. Early work using functional magnetic resonance imaging (fMRI) identified states differentiating pictures and sentences, or whether individuals were reading words describing food, people, or buildings (Mitchell et al., 2004). This work has been extended to other states relating to mental workload. Several researchers have used machine learning to classify task difficulty levels with 80% or better accuracy within an individual (Wilson and Russell, 2003—EEG with ANN; Gateau et al., 2015—fNIRS with SVM). Zander et al. (2017) used this method during surgery simulations with secondary auditory tasks (EEG with LDA), while others used a similar process in an ecological simulator and real flight settings (Gateau et al., 2018—fNIRS with SVM). Wilson and Russell (2007) used electroencephalography to implement automation aiding in a UAV navigation task, which led to greater performance particularly when the classifier was trained within individuals (EEG with ANN). Similar studies have deployed automation under high-load conditions during air traffic control (Aricò et al., 2016—fNIRS with stepwise LDA). While most tasks have been classified at two or three mental workload levels, there has been evidence of success at up to seven levels in complex supervisory environments (Zhang et al., 2016—EEG with ECNN). Hong et al. (2015) successfully used fNIRS to discriminate between different tasks such as mental arithmetic versus motor imagery and on-task versus off-task performance (fNIRS with LDA).

Most classifiers have been effective within participants. It has also been shown that by accounting for the hierarchical nature of human-time series data, classifiers can be trained at the group level with similar individual performance to those trained directly at the individual level (Wang et al., 2011—EEG with Hierarchical Bayes). Although unsuccessful in Wang et al. (2011), neural networks have also been successful in classifying non-linear characteristics of neurological data to create cross-person classifiers (Stikic et al., 2014). Unfortunately, even with the past successes of within task difficulty level classification, cross-task classification remains a technical challenge (Baldwin and Penaranda, 2012—EEG with ANN). However, there has been success in directly testing near transfer within an n-back task where the kind of stimuli remembered (i.e., letters, images, or locations) was the primary manipulation (Grimes et al., 2008). Others have also shown indirect cross-task transfer *via* improving task performance with an adaptive control algorithm or associating an algorithm with subjective workload measures (Yuksel et al., 2016).

Understanding individual differences in mental faculties are of key importance to understanding mental workload states or other performance concepts. This is explicit in theories such as perceptual load theory (Lavie et al., 2004), cognitive load theory (Sweller, 1994), and multiple resource theory (Wickens, 2008). All refer to a mental faculty (i.e., WM, attention, etc.) that has a limit, and beyond that limit, mental states and, relatedly, performance change. Yet, these limits are not dictated by the task; they are dictated by the individual. It is well understood that individuals differ in terms of WM, attention reaction time, perceptual acuity, etc. and these differences are what define changes in states of mind. This means that conditions that might elicit a state of mental overload in one individual may elicit a state of adequate load in another, and potentially even a state of underload in a particularly gifted individual. Further considering work in mental resource and perceptual load theory, individuals can easily differ in the overall parameters that define an overload state. It is easy to imagine a situation when one individual reaches a state of mental overload due to the attentional demands of a task exceeding their capacity, but the same task doesn’t exceed their capacity for WM, or vice versa.

Providing key trait level individual differences to the classification algorithm could increase the performance of supervised statistical learning classifiers. The common practice is to rely on task parameters to classify difficulty, which a classifier uses to learn. Using task parameters is the standard because it is assumed that they provide a more objective label to train over. However, this assumption only stands if we are using the algorithm to differentiate between task conditions, essentially task decomposition. This is not the case during workload classification, as we have already argued that mental states of workload are not wholly defined by the conditions of the task but are also sensitive to individual differences. There are multiple methods that can be used to incorporate individual differences into labeling training data for supervised statistical learning.

Stress–strain curves and Rasch models can account for individual differences by including a capacity metric and applying logical rules to the model. A stress–strain model (Ramberg and Osgood, 1943; Mander et al., 1988) has levels of stress modeled along the *x*-axis and some measure of a material’s reaction to that stress on the *y*-axis. The model has an initial linear phase where reaction builds with increasing stress, eventually asymptotes, and finally begins to decline. This model can be adapted to the context of mental workload classification by using task difficulty in place of stress and performance in place of reaction (for details, see the section *Stress–Strain Curve*). In contrast, Rasch models (Andrich, 1988) plot the probability of successfully responding to a task against the task’s difficulty. These curves can be created for each individual such that the curve’s intercept represents their scoring on the trait that is responsible for the difficulty of the task (for details, see the section *Rasch Model*).

The present experiment had three aims with regard to applications in neuroadaptive systems. First, test the efficacy of different models of labeling mental workload states for supervised machine learning. Second, compare the efficacy of regularized regression methods to ensemble classification methods. Third, compare labeling and classification techniques across two different memory-based tasks. We hypothesized incorporating individual differences and states at the point of data labeling would improve performance for initial training and cross-validation, for cross-participant transfer, and for cross-task transfer.

## Materials and Methods

### Participants

Thirty-four Northrop Grumman Corporation (NGC) employees volunteered to participate in this experiment. Recruitment efforts included a solicitation in a company newsletter and word of mouth. All participants’ behavioral and neurophysiological data were de-identified. The only requirement to participate was that the participant was 18 years of age or older and an employee of NGC. One participant did not understand the task instructions and was unable to adequately perform the task; this person was removed from the sample, leaving a final sample size of 33 participants. Nine of the 33 participants were female (27.27%): the rest, male. Participants’ ages ranged from 18 to 65 (*M* = 29, *SD* = 10.5). Participants’ highest level of education achieved was evenly distributed across GED, Bachelors, and Master’s degrees (30.3%), with the remainder being two participants with associates degrees and one with a Doctorate. This research complied with the American Psychological Association Code of Ethics and all participants provided informed consent prior to participation. The study was approved by Advarra’s External Institutional Review Board.

### Materials

#### Intelligence, Surveillance, and Reconnaissance Task

##### Fnirs baseline calibration task

An fNIRS baseline calibration task and an ISR task were programmed using Qt (with a combination of C++ and JavaScript) and compiled together into an executable software program. The baseline calibration task for fNIRS imaging was created to elicit consistent brain activity by introducing a stimulus that did not require intense mental processing. Instead of the typical approach, which is to collect baseline brain activity without a stimulus or instructions, our method attempts to control the mental states during baseline across participants by eliminating possible variability from mind wandering. The stimulus was a black fixation cross on a gray background that moved smoothly along a black line in the shape of a bowtie. The fixation cross starts at the upper left corner of the bowtie and moved for a duration of 6 s, during which the cross passed over the entire bowtie figure before ending at the center.

##### ISR task description

**FIGURE 1:**
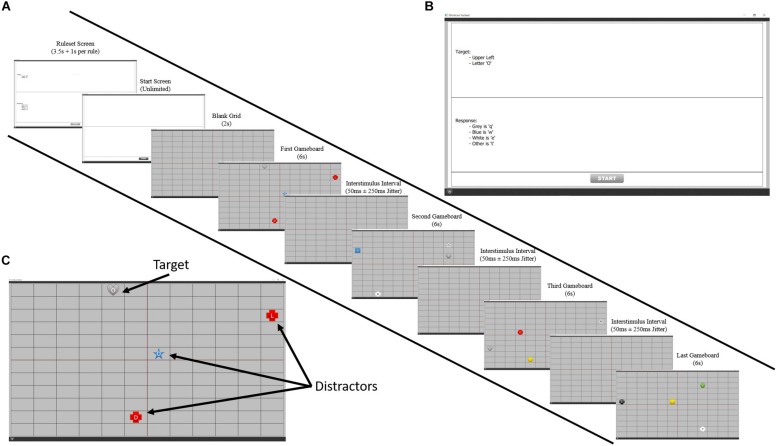
A visual representation of the ISR task. **(A)** Progression of an example ISR level. Participants were presented with a ruleset for that level on the first screen which disappeared after a short duration. Participants had to remember these provided rules throughout the entire level for accurate target identification and response. After pressing the start button on the following screen, they would play four boards using this ruleset. **(B)** An example of a level ruleset. **(C)** Example of an ISR board. On each board, participants saw four stimuli and had to use the mouse to select the target based on the target identification rules and press the appropriate letter on the keyboard based on the target response rules. Based on the ruleset shown in panel **(B)**, a correct response would be to click gray heart with an “O” and pressing the letter “q” on the keyboard.

This task was created to simulate the basic components of ISR. For each level, the participant was presented with a set of rules that contained information necessary for identifying and responding to a given target. After a set period of time, the rules disappeared and a screen consisting of a grid with four quadrants was presented. On the grid, groups of four stimuli would appear, one of which was the target. Using the information from the rules that the participant was maintaining in their WM, the participant had to visually search for the target on the grid and identify it by clicking on it with the mouse pointer. Once the participant clicked on the target, they then had to make a correct keyboard response by remembering the response rules and pressing a specific key. See Figure 1 for an example of a level.

The stimuli presented on the grid consisted of colored shapes with a letter in the center. These objects could appear with any combination of the following four features: color (blue, red, yellow, green, white, black, and gray), shape (circle, triangle, square, pentagon, hexagon, star, and hear), letter (l, t, b, d, c, o, m, and w) and quadrant (upper left, upper right, lower left, and lower right). There were a total of 1,568 possible combinations of these four features, each being a unique stimulus. A randomized list was used to select the four pieces to appear on the grid for each board and then this was hardcoded so that every participant saw the same levels. The response keys could be q, w, e, r, t, or no response and which key needed to be pressed depended on a rule based on a feature of the target (i.e., target is blue = “q,” target is red = “w”).

Difficulty was manipulated through changes in memory load by varying the number of rules that the participant had to maintain in WM in order to successfully complete a level. The number of rules that had to be maintained ranged from 1 to 10, resulting in 10 levels of difficulty. Each participant had to complete three blocks of trials. Each block consisted of 10 levels, one for each difficulty, with each level containing four groups of stimuli.

The duration of the rule-screen varied with the amount of rules being presented. This was done in an attempt to ensure that the time a participant viewed the rule-screen was spent reading text rather than rehearsing the rules, which would aid encoding and lessen the load on WM. The rule-screen’s duration started at 2 s and had an additional 1.5 s added for every rule. Thus, the shortest duration for a rule-screen was 3.5 s for level 1: the longest, 17 s for level 10. After this duration, the rules would disappear, leaving a white screen with a “start” button at the bottom, allowing the participant to start the level when ready. The first group of stimuli within each level appeared 2 s after the play button was clicked, with a jitter. Each group was presented for 6 s before disappearing, during which the participant could find, click the target, and then press the correct response key. The entry time for each subsequent group within a level was 50 ms after the disappearance of the previous group, also with a jitter, but ensuring that two boards would not appear at the same time. The jitter times were selected by randomly sampling times from Gaussian distribution with a mean of 0 and a standard deviation of 0.16, which ensures that 99.7% of the values sampled will be in the interval [-250 ms, 250 ms]. Based on how the participant responded to the group of stimuli, they received a score. For clicking the target, they received a base score of five points and then additional points based on how quickly they clicked. For example, if they clicked the target after 3 s, they would receive 5 (base score) + 6 s (group duration) – 3 s (click time) = 8 points. The score for the keyboard response was calculated similarly: 5 points + board duration - key press speed. These were summed to get a total score. If only a click was made and not a key response, then only the click score was used. Finally, the total score was multiplied by a difficulty factor, which was equivalent to the number of rules in the level. The purpose of this was to give more credit for successfully completing harder levels. These scores were not displayed to the participants, in order to discourage random responses in an attempt to score points. Target and response scores were calculated only for the first instance of each event—i.e., if a target was clicked more than once, only the first click event resulted in a score. Any event occurring on a non-target, distractor, resulted in no score.

##### ISR task pilot testing

An initial version of the ISR task was created, in which each difficulty level had three versions, created by varying the number of target and response rules. For example, difficulty level 5 had the following versions: three identification and two response rules, two identification and three response rules, and one identification and four response rules. We did a pilot test of these 30 levels, with three trials for each, where we collected data from five participants. We attempted to fit a quadratic polynomial function to the results of the pilot test to select the versions of each level that best fit that model. A quadratic function was used to represent a stress–strain curve with a defined asymptote. During fitting, we observed that there were three levels that did not have a version that fit the model well. We also found that the number of response rules played a bigger part in the task’s actual difficulty (measured by participant performance) than the number of target identification rules. With this knowledge, we created six new levels and performed a second round of pilot testing, again consisting of five participants with three trials per level. With the results from the second pilot test, we selected the 10 levels that had the strongest fit to a quadratic polynomial function.

#### Spatial WM Task

In a previous experiment, 30 different participants from a large, mid-Atlantic university completed a spatial WM dot task on a computer. The de-identified data were used, with permission of the authors, to assess the generalizability of the methods developed in this paper. In the WM task, participants completed 10 trials at each of 10 difficulty levels, corresponding to the number of dots they were asked to remember (1 to 10 dots). This resulted in a total of 100 trials that were randomly presented. During each trial, participants saw a black screen for 8 s, followed by a white fixation cross on a gray background for 1 s. Then, the randomly spaced dots appeared for 1 s, followed by a random 4-s noise mask. Participants responded by using a mouse to click where they remembered the dots being located on a gray response screen with a white fixation cross. When they were done selecting locations with the mouse, they pressed the space bar to move to the next trial. For full task details and results, see the full article by McKendrick and Harwood (under review).

#### fNIRS Device

Measurements of PFC hemodynamics were acquired *via* raw light intensities through an fNIRS Devices fNIR 2000 S system (fNIR Devices LLC, Potomac, MD, United States^1^) composed of four light emitters and eight photodetectors. This configuration yields 16 dual-wavelength optodes measuring near-infrared wavelengths of 730 and 850 nm. The sensor was placed over the forehead in order to image prefrontal cortical regions. The imaging temporal resolution was 10 Hz and the average emitter to detector distance was 2.5 cm, allowing for light penetration of approximately 1.25 cm deep into the human head. COBI Studio software was used for data acquisition and visualization of data quality during imaging (Ayaz et al., 2011).

### Procedure

At the start of the study, participants were seated at a desk in a quiet room in front of a Dell computer system that ran both the ISR task and the fNIRS data acquisition software. Dual 21-inch monitors were used, one to display the fNIRS data stream to the experimenter and the other for the participant to complete the ISR task. Participants used a mouse and keyboard to interact with the ISR task. The experimenter followed a printed experimental protocol that contained an ordered procedure and a script to read to each participant to keep sessions between participants as similar as possible. After participants provided written informed consent and completed a demographic survey, the fNIRS headband was placed around their head and was secured using self-adhesive bandaging strips to reduce motion artifacts and ambient light. Noise canceling headphones were placed over the participant’s ears and the fNIRS device was started for a visual assessment of the quality of the data. Any necessary adjustments were made at this point.

Once the fNIRS data quality was determined to be sufficient, data recording was started and the ISR task was launched. All instructions were built into the software and presented *via* text on the screen. Before starting the ISR task, participants could take as much time as was necessary for them to understand the instructions. Once they felt they understood how to perform the task, they were presented with three practice levels of varying difficulty. These levels had two, six, and eight rules, respectively. Different from the regular task, the practice groups of stimuli would not disappear until participants clicked the correct target and pressed the correct response key. Finally, following reminder text about the rules of the task, the task began. As mentioned above, the trials were split into three blocks, where each level was seen once within a block. Each level had four groups of stimuli (or trials). Therefore, each participant saw 120 trials over, approximately, 30 min.

### Data Analyses

#### fNIRS Data Preprocessing

The fNIRS processing pipeline was created using Python (Python Core Team, 2015). Each participant’s raw light intensities were first motion corrected using the procedure outlined by Cooper et al. (2012), Molavi and Dumont (2012), and Brigadoi et al. (2014). We used a Daubechies 5 (db5) wavelet filter from the PyWavelets/pywt package to compute the wavelet coefficients and applied a threshold, α, of 0.1, the same used by Molavi and Dumont (2012). Then, the data were low-pass filtered using a cutoff frequency of 0.12 Hz using the SciPy.signal package (Jones et al., 2001) to attenuate high-frequency noise associated with respiration and cardiac cycle effects (Ayaz et al., 2010). Data were then converted to optical density (OD) by calculating the -log of the filtered signal divided by the average, filtered signal from the calibration task described in the section *fNIRS Baseline Calibration Task*. Deoxygenated hemoglobin (Hb) and oxygenated hemoglobin (HbO_2_) levels, in relation to the baseline levels, were calculated by submitting the filtered light intensities to the modified Beer-Lambert law (Ayaz et al., 2012a, b). We then used the calculated Hb and HbO_2_ values to calculate the oxygenation levels (Oxy) as HbO_2_ - Hb and the approximate percent change in blood volume (HbT) as HbO_2_ + Hb. We also calculated quadratic (*y* = *x* + *x*^2^) and cubic (*y* = *x* + *x*^2^ + *x*^3^) polynomials of each hemodynamic measure (Hb, HbO_2_, HbT, and Oxy). We included these polynomials because previous research (McKendrick and Harwood, under review) has shown that participants’ hemodynamic response is non-linear as they transition through mental workload states (i.e., underload and overload). Given that the ISR task primarily taxed the same underlying neural mechanism—WM—we suspected the hemodynamic response to the ISR task would follow the same pattern and wanted to include these features for our models.

#### Behavioral Data Analyses

##### Generalized and linear mixed-effects models

All forthcoming descriptive modeling tests employ general linear models, linear mixed-effects models, or generalized linear mixed-effects models implemented in R (R Core Team, 2013) *via* the lme4 package (Bates et al., 2014). Linear mixed-effects estimates were computed with restricted maximum likelihood and generalized linear mixed estimates were computed with maximum likelihood and binomial link functions. Denominator degrees of freedom and *p*-values were estimated *via* Satterthwaite corrections implemented *via* the lmerTest package (Kuznetsova et al., 2017).

##### Nested model comparison

The Bayesian information criterion (BIC; Schwarz, 1978) was used to select the fixed and random effects in the final models for each dependent variable. Competing models were constructed by adding potentially meaningful random and fixed effects to a null model. The null model was specified in each case as having no fixed effects and a random effect of participant intercept. The competing models were compared with BIC and the strength of evidence criterion described by Kass and Raftery (1995) was employed. In the procedure, deviations of greater than 2 BIC are viewed as a meaningful difference. The final model was selected based on having the lowest BIC, with no other models of interest having a BIC deviance of less than 2 (McKendrick et al., 2017).

#### Behavioral Data Labeling Methods

We investigated three methods of labeling mental workload based on participants’ performance on the ISR and WM tasks. We compared a split based on task difficulty (difficulty split), Rasch modeling that is based in item response theory, and a stress–strain relationship adapted from physics. Using the different labeling techniques, we were able to label each sample of fNIRS data as being measured while the participant was in one of three mental workload states: underload, adequate load, or overload. In the following sections, we provide an overview of each labeling technique.

##### Difficulty split

**FIGURE 2:**
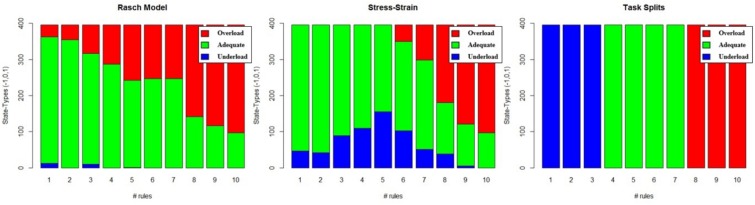
Example breakdown of classification under each of the three labeling methods. **(Left)** Rasch model, **(Middle)** Stress–strain, and **(Right)** Difficulty Split.

The most common method for labeling workload states is to use a task’s difficulty conditions. We refer to this method as difficulty split labeling. For this method, at least one condition is designed to be more challenging than another. Essentially, a task parameter, such as number of items, is used to categorize difficulty. If there are many difficulty conditions, as in our experiment (i.e., 10 difficulty levels), the conditions can be grouped to reduce the number of class labels. We grouped our difficulty conditions into three classes for labeling. The underload class referred to conditions with 3 or less rules, the adequate load class refers to conditions with 4 to 7 rules, and the overload class referred to conditions with 8 to 10 rules (1). For a visual representation, see the left panel in Figure 2.

##### Rasch model

This capacity model is based on adopting Item Response Theory: Rasch Models, as a person–characteristic curve, for identifying individual state capacities. A Rasch model assumes variability in either a latent trait or the content of a group of test items. The varying condition is modeled on the *x*-axis and the probability of successes is modeled on the *y*-axis. The function for representing this response is logistic in nature, it has clearly defined upper and lower bounds, and its parameters consist of an intercept and slope. This model can be adopted in the context of mental workload classification by using test difficulty on the *x*-axis and the probability of correct response on the *y*-axis. A curve can be fit to each individual that participates in the test with mixed-effects modeling. For this model, we assume that an individual’s latent trait is synonymous with their capacity for that mental faculty. If we include information on an individual’s performance at their capacity, which is the intercept of the Rasch model (i.e., point of 50% success), we can create a simple set of rules for defining the states of underload, adequate load, and overload (see the section *Mental State Labeling Definitions for Capacity Models* for an example).

##### Stress–strain curve

This capacity model is based on adopting stress–strain curves, in a general sense, for identifying individual state capacities. A stress–strain model assumes some level of stress modeled on the *x*-axis and some measure of performance/reaction to that stress on the *y*-axis. The model has an initial linear phase where performance builds with increasing stress, eventually asymptotes, and finally begins to decline. This model can be adopted in the context of mental workload classification by using test difficulty in place of stress on the *x*-axis and a performance metric that has a weighting parameter from test difficulty on the *y*-axis. Like with Rasch models, a curve can be fit to each individual that participates in the test with mixed-effects modeling. Also similar to Rasch models, we can use an estimate of an individual’s latent trait capacity (*x*,*y*-coordinate for stress–strain stationary point/asymptote) to define states of underload, adequate load, and overload (see the section *Mental State Labeling Definitions for Capacity Models* for an example).

##### Mental state labeling definitions for capacity models

Rasch and stress–strain models both have explicit representations of capacity. We use this representation to define different mental workload states. After the model has been built, a task is compared to an individual’s capacity to perform the task in order to determine the mental workload state the individual was in. If the task’s difficulty is greater than the individual’s capacity and their performance on the task is below the modeled performance, the state is labeled as overload. Conversely, if the task’s difficulty is less than the individual’s capacity and their performance on the task is below the modeled performance, the state is labeled as underload. In all other conditions, the state is labeled as adequate load.

For example, a participant was modeled and found to have a capacity of 5, at which level they were expected to achieve a score of 15. They actually performed a task with a difficulty of 7 and received a score of only 10. This task exceeded their capacity, 5, and their performance was below their expected performance of 15; therefore, this condition would be labeled as overload. Had their performance been 15 or greater at this capacity, this condition would have been labeled as adequate load.

Continuing the same example, the individual also performed a task with a difficulty of 4 and received a score of 10. The individual’s model predicted they would score a 12 on a task with difficulty of 4. Therefore, they were below their capacity of 5 and their actual performance, 10, was below their expected performance of 12; therefore, this condition would be labeled as underload. Had their performance been 12 or greater, this condition would have been labeled as adequate load.

**FIGURE 3:**
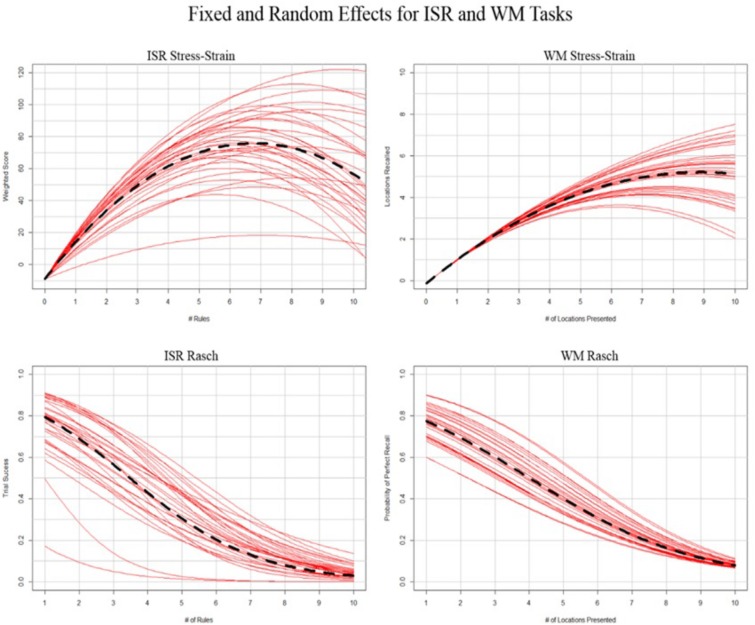
Representation of general **(left)** and generalized **(right)** mixed effects regressions. Black hashed line depicts fixed effects, and red lines depict random effects conditioned on individual participants.

Following the above procedure, we created a mixed-effects model representing the population estimates of performance as well as estimates for each individual; this is depicted in Figure 3 for Rasch and stress–strain models. By applying the above rules to the individual conditioned models from Figure 3, we created labels specific to each individual. Summing the labels (-1 = underload, 0 = adequate load, and 1 = overload) and plotting those sums against each difficulty level produced Figure 2, which represents the distribution of labeled states for each of the three labeling methods.

#### Data Balancing Techniques

The Rasch and stress–strain labeling techniques result in large class imbalances, as can be seen from Figure 2, while the difficulty split technique results in fairly balanced classes. There are several techniques used to handle unbalanced datasets, such as collecting more data, modifying class weights, or over- and under-sampling techniques. Collecting enough data to result in a balanced dataset is infeasible, so we explored both modifying class weights while training and balancing the datasets beforehand. Modifying class weights is done during model training and was added as a parameter to explore in our models’ optimizations (see the sections *Elastic Net Optimization* and *Random Forest Optimization*). The over-sampling technique we used was the Synthetic Minority Over-sampling Technique (Smote). Over-sampling with Smote helps deal with the class imbalance issue but it can cause the borders between the classes to blur, making the classification problem more difficult. In order to deal with this, we used two under-sampling techniques as cleaning methods after our Smote over-sampling: Tomek links and Edited Nearest Neighbor (ENN) (Batista et al., 2004). Below, we provide a brief overview of these techniques. Before the data balancing methods were applied, we separated three participants’ data for each task. This creates a three-participant validation set for each task and ensures that this validation set data does not bleed into the training set due to the data balancing.

##### Synthetic minority over-sampling technique

SMOTE is an over-sampling method that creates new minority class examples *via* interpolation. The main idea is to find groups of minority class samples that lie near one another and to interpolate between them, thus creating new, synthetic samples of the minority classes. This allows for the minority class to be increased without leading to over-fitting, as occurs when over-sampling with replacement. The technical details of how the Smote algorithm performs can be found in Chawla et al. (2002).

##### Tomek links

A Tomek link is defined as two data points that are each other’s closest neighbors but have different class memberships (Tomek, 1976). If this is the case, then we can argue that, either one of these points is noise or that both are borderline samples. Once a Tomek link has been found, we can either remove the point belonging to the majority class or we can remove both points. The first would be an under-sampling technique, while the second is a data cleaning technique. In our case, we are interested in using Tomek links as a data cleaning technique as removing both points will most increase the class separation. Thus, we first apply the Smote algorithm to our original dataset and then use Tomek links to clean the oversampled dataset, resulting in a balanced dataset with well-defined class borders, as suggested by Batista et al. (2004). To implement the Smote-Tomek method of over-sampling followed by cleaning, Python’s SMOTETomek class from the imblearn library was used. Using the Smote-Tomek methodology increased the number of samples by 65.21, 5.66, and 65.66% for the stress–strain, difficulty split, and Rasch model labeling techniques, respectively. This makes sense, when referring to Figure 2, where we see that the difficulty split technique already has a nearly even class split and the other two methods are drastically imbalanced. With the increased number of samples described above, all three labeling techniques have approximately even class splits.

##### Edited nearest neighbors

As suggested by Batista et al. (2004), we also explored using Wilson’s ENN rule as a data cleaning technique after applying the Smote algorithm (Wilson, 1972). The ENN rule looks at each data point, finds its three nearest neighbors, and compares their class labels. If the data point’s class differs from at least two of its nearest neighbors, then that data point is removed. To implement the Smote-ENN method of over-sampling followed by cleaning, Python’s SMOTEENN class from the imblearn library was used. Using the Smote-ENN methodology increased the number of samples by 64.63, 5.28, and 65.20% for the stress–strain, difficulty split, and Rasch model labeling techniques, respectively. Again, this makes sense for the same reasons mentioned in the section *Tomek Links*. With the increased number of samples described above, all three labeling techniques have approximately even class splits.

#### Machine Learning Algorithms

We investigated two algorithms for classifying mental workload based on the fNIRS signal. We compared a regularized regression model (i.e., elastic net) (Zou and Hastie, 2005) to an ensemble discursive model (i.e., random forest; Breiman, 2001). These models were chosen for their interpretability and, typically, good generalizability, as well as their Python implementations available *via* the scikit-learn library. Below, for each of the two algorithms, we detail the parameters over which we explored during model optimization. Each technique was optimized for the area under the curve (AUC) of the model’s receiver operating characteristic curve (ROC; TP rate vs. FP rate). ROC AUC was chosen as our optimization parameter because, as compared to accuracy, it provides a more complete single metric of model misclassifications. For both models, we used all of the hemodynamic parameters we computed (Hb, HbO_2_, HbT, and Oxy) and all of their polynomial formations (see the section *fNIRS Data Preprocessing*) across all 16 channels as features and all measurements taken during the task (ISR or WM) as samples. All optimizations, as described in the following sections, were performed on the training sets of data for each task only; the optimizations were not performed using the three-participant validation sets set aside before data balancing (see the section *Data Balancing Techniques*).

##### Elastic net optimization

We optimized three tuning parameters: the mixing-ratio between L1 and L2 regularization, the strength of penalization, and the misclassification penalization weights associated with the classes. The mixing-ratio controls the type of penalty applied and is bound between zero and one. A mixing-ratio of zero corresponds to an L2, ridge penalty: one corresponds to an L1, LASSO penalty. The penalization parameter controls how strong the penalization is; the stronger the penalization, the more regularization of the model’s feature coefficients, potentially resulting in a sparse model. The class weights control how much a misclassification for each class is penalized and can be used to help account for class imbalances in the dataset by applying greater penalizations to the minority classes; this is the other method of dealing with imbalanced datasets mentioned in the section *Data Balancing Techniques*.

The optimization was performed using a grid-search cross-validation method. The grid spanned 20 mixing-ratio values linearly spaced between (0, 1.0), inclusive, 40 penalization values logarithmically spaced between (10^–2^, 10^1^), inclusive, and three class weight settings of [None, “balanced,” {-1: 100, 0: 1, 1: 100}]. The “None” class weight setting does not apply any misclassification penalization, while the “balanced” setting sets the weights to be inversely proportional to the class frequencies. The final setting sets the class weight for each minority class (underload and overload) to 100 times greater than the majority class (adequate load), thus pushing the model to better learn the minority classes. Each of the 2,400 parameter combinations was evaluated using stratified fivefold cross-validation with ROC AUC as the evaluation metric. During the cross-validation, we did not place any further restraints on how the training data were split into the folds other than it be stratified, to ensure that each fold was a good representation of the whole in terms of class balance.

##### Random forest optimization

We optimized four tuning parameters: the minimum number of samples required to split an internal node, the number of features to consider when looking for the best split, the number of trees in the forest, and the misclassification penalization weights associated with the classes. The minimum number of samples required to split a node controls how large each tree can grow. The lower the number, the deeper the tree can grow, with two resulting in fully developed trees. The number of features to consider sets the size of the random subset of features considered when splitting a node. The lower the number, the more variance is reduced but bias is also increased. The number of trees in the forest controls how many decision trees the forest contains. More trees is generally better, but is limited by computation time and performance plateaus as the number increases. The class weight functions the same as for the elastic net described in the section *Elastic Net Optimization*.

The optimization was performed using a grid-search cross-validation method. The grid spanned the following values for each parameter: four minimum number of samples to split values: [2, 5, 10, 50], three maximum number of features to consider settings: [“sqrt,” “log2,” 0.50], two number of trees in the forest values: [50, 100], and two class weight settings: [None, “balanced”]. The “sqrt” and “log2” settings for the maximum number of features take that function of the total number of features available [i.e., *sqrt(total_features)*, *log_2_(total_features)*], and the decimal value takes that percentage of the total features. The class weight settings are, again, as described for the elastic net in the section *Elastic Net Optimization*. Each of the 48 parameter combinations were evaluated using stratified fivefold cross-validation (with the same requirements as above) and ROC AUC as the evaluation metric. There were fewer models tested for the random forest than for the elastic net because the random forests take significantly longer to train and test.

#### Overall Processing and Optimization

Through the combination of different labeling techniques (difficulty split, Rasch, and stress–strain) and class balancing techniques (unbalanced, Smote-Tomek, and Smote-ENN), we had nine different datasets for each task (ISR—see the section *Intelligence, Surveillance, and Reconnaissance Task* and WM—see the section *Spatial WM Task*). For each dataset, we optimized two algorithms (elastic net and random forest), resulting in 36 optimized models. To validate the models, we performed two tests. First, we held out three participants from each task’s training dataset to test cross-person transfer of each model (i.e., a model optimized on ISR data was validated against hold-out participants’ ISR data). Next, the models were used to classify the data from the other task to test cross-task transfer of each model (i.e., a model optimized on ISR data was tested on WM data). In this way, we were able to gauge a model’s performance on both a new participant doing the same task the model was trained on, as well as a new participant performing a new task.

## Results

### Behavioral Results

#### Isr Task Analysis

##### Irs stress–strain labeling

The most parsimonious stress–strain model for the ISR task score, as defined by the methods described in the sections *Stress–Strain Curve* and *Mental State Labeling Definitions for Capacity Models*, specified a polynomial quadratic effect of number of rules (i.e., intercept, *B* = –8.99, SE = 2.02, df = 3893, *p* = 8.47e–6; linear slope, *B* = 25.05, SE = 1.25, df = 81.51, *p* = 2e–16; quadratic slope, *B* = –1.85, SE = 0.12, df = 60.35, *p* = 2e–16). Random effects of participant’s linear and quadratic slopes for number of rules were also selected.

The random effect of participant’s linear slope suggests that individuals differed in the initial load of their memory for the task rules. The random quadratic slope suggests that individuals also differed in how their memory systems responded to being overload by the task rules (Figure 3, top left). However, after accounting for this random variance, there was still a parsimonious polynomial quadratic fixed effect of number of rules. Using this model, we labeled the participants’ mental workload state throughout their performance on the ISR task.

##### ISR rasch labeling

The most parsimonious Rasch model for the ISR task accuracy, as defined by the methods described in the sections *Rasch Model* and *Mental State Labeling Definitions for Capacity Models*, specified a linear effect of number of rules (i.e., intercept, *B* = 3.48, SE = 0.30, *p* = 2e–16; linear slope *B* = –0.54, SE = 0.04, *p* = 2e–16). Random effects of participant and linear slope for number of rules were also selected.

The random effect of participant suggests that individuals differed in their capacity for task performance. Furthermore, the probability of successful performance was also influenced by the number of rules presented (Figure 3, bottom left). However, after accounting for this random variance, there was still a parsimonious fixed effect of number of rules. Using this model, we labeled the participants’ mental workload state throughout their performance on the ISR task.

#### WM Task Analysis

##### WM stress–strain labeling

The most parsimonious stress–strain model for the WM number of locations recalled, as defined by the methods described in the sections *Stress–Strain Curve* and *Mental State Labeling Definitions for Capacity Models*, specified a polynomial quadratic effect of number of locations presented (i.e., intercept, *B* = –0.14, SE = 0.09, df = 2970, *p* = 0.115; linear slope, *B* = 1.20, SE = 0.04, df = 2970, *p* = 2e–16; quadratic slope, *B* = −0.07, SE = 0.01, df = 187.40, *p* = 2e–16). Random effects of participant’s quadratic slope for number of locations were also selected.

The random effect of participant’s quadratic slope suggests that individuals differed in how their memory systems responded to being overloaded by the number of locations to be recalled (Figure 3, top right). However, after accounting for this random variance, there was still a parsimonious polynomial quadratic fixed effect of number of locations. Using this model, we labeled the participants’ mental workload state throughout their performance on the WM task.

##### WM rasch labeling

The most parsimonious Rasch model for the WM response accuracy, as defined by the methods described in the sections *Rasch Model* and *Mental State Labeling Definitions for Capacity Models*, specified a linear effect of number of locations to be recalled (i.e., intercept, *B* = 4.01, SE = 0.19, *p* = 2e-16; linear slope, *B* = −0.41, SE = 0.01, *p* = 2e–16). Random effects of participant and linear slope for number of locations were also selected.

The random effect of participant suggests that individuals differed in their capacity for task performance. Furthermore, the probability of successful performance was also influenced by the number of locations presented (Figure 3, bottom right). However, after accounting for this random variance, there was still a parsimonious fixed effect of number of locations to be remembered. Using this model, we labeled the participants’ mental workload state throughout their performance on the WM task.

### Model Optimization Across All Parameters

Our analyses were performed to address the following hypotheses regarding incorporating individual differences into class labeling: (1) this would lead to better initial training and cross-validation of supervised machine learning algorithms, (2) this would lead to better performance of an algorithm trained on person “A” and used to predict the states of person “B,” and (3) this would lead to better performance of an algorithm trained on task “A” and used to predict the states of persons performing task “B.” The sections *ISR Cross-Validation* and *WM Cross-Validation* discuss the tests of hypothesis 1, the sections *ISR Hold-Out Participants Validation* and *WM Hold-Out Participant Validation* discuss the tests of hypothesis 2, and the sections *ISR Transfer to WM Validation* and *WM Transfer to ISR Validation* discuss the tests of hypothesis 3.

After, we optimized the 36 models described in the section *Overall Processing and Optimization*, we performed six linear regressions to test the effects of manipulating labeling, algorithms, and class balancing. To improve the interpretability of our null hypothesis, the ROC AUCs were converted to GINI Index values using the relation GINI = AUC^∗^2-1. Therefore, the null hypothesis in our regressions represent a true zero of no information learned by the model. Our random forest model trained on the Smote-Tomek balanced dataset with Rasch labels was our best-performing model during optimization cross-validation for both the ISR and WM datasets. Therefore, we Dummy coded these parameters in our regression so we could compare other labeling, algorithms, and class balancing performance against this model.

Tables 1–6 present the results of each test as well as the results of our regression across the models. In each table, the “Effects” column shows the model tested, in the format “Labeling technique: Algorithm: Balancing technique,” the “*B* Estimate GINI” column shows the shifted GINI Index unstandardized beta coefficient as described above, and the “Std. Err.” and “*p*” columns show those parameters for each unstandardized beta. Tables 1, 4 present the results of the optimization process for each model on the ISR and WM datasets, respectively; in these tables, the “AUC” column is the mean ROC AUC across the cross-validations performed for the most optimal model parameters. Tables 2, 5 present the results of the in-task, cross-person test for the ISR and WM datasets, respectively; in these tables, the “AUC” column is the mean ROC AUC the model achieved when predicting the mental workload state of the validation participants. Finally, Tables 3, 6 present the results of the cross-task test for the ISR and WM datasets, respectively; in these tables, the “AUC” column is the mean ROC AUC the model achieved when predicting the mental workload state of the other task’s participants.

**TABLE 1:** ISR cross-validation.

**Effects**	**AUC**	***B* Estimate**	**Std.**	***p***
		**GINI**	**Err.**	
**Rasch: Random-Forest: Tomek**	0.91	0.82	0.03	**3.57E−37**
Stress–Strain: **Random-Forest: Tomek**	0.87	–0.07	0.05	1.20E−01
Diff-Split: **Random-Forest: Tomek**	0.82	–0.17	0.05	**4.08E−04**
**Rasch:** Elastic Net: **Tomek**	0.77	–0.27	0.05	**1.21E−07**
**Rasch: Random Forest**: unbalanced	0.74	–0.34	0.05	**3.36E−10**
**Rasch: Random Forest:** ENN	0.91	0.00	0.05	9.80E−01
Stress–Strain: Elastic Net: **Tomek**	0.64	–0.20	0.07	**3.17E−03**
Diff-Split: Elastic Net: **Tomek**	0.54	–0.31	0.07	**1.54E−05**
Stress–Strain: **Random Forest:**	0.64	–0.14	0.07	**3.79E−02**
Unbalanced				
Diff-Split: **Random Forest:** Unbalanced	0.50	–0.31	0.07	**9.25E−06**
Stress–Strain: **Random Forest:** ENN	0.88	0.00	0.07	9.55E−01
Diff-Split: **Random Forest:** ENN	0.83	0.00	0.07	9.71E−01
**Rasch:** Elastic Net: Unbalanced	0.56	–0.09	0.07	1.66E−01
**Rasch:** Elastic Net: ENN	0.78	0.01	0.07	8.35E−01
Stress–Strain: Elastic Net: Unbalanced	0.64	0.58	0.09	**2.36E−08**
Diff-Split: Elastic Net: Unbalanced	0.53	0.74	0.09	**1.57E−11**
Stress–Strain: Elastic Net: ENN	0.64	–0.02	0.09	8.36E−01
Diff-Split: Elastic Net: ENN	0.54	0.00	0.09	9.60E−01

*Bold value is *p* < 0.05.*

**TABLE 2:** ISR hold-out participants.

**Effects**	**AUC**	***B* Estimate**	**Std.**	***p***
		**GINI**	**Err.**	
**Rasch: Random Forest**	0.81	0.62	0.06	**2.79E−13**
Stress–Strain: **Random Forest**	0.67	–0.28	0.09	**2.82E−03**
Diff-Split: **Random Forest**	0.51	–0.60	0.09	**1.16E−08**
**Rasch:** Elastic Net	0.53	–0.56	0.09	**7.02E−08**
Stress–Strain: Elastic Net	0.58	0.37	0.12	**4.50E−03**
Diff-Split: Elastic Net	0.49	0.53	0.12	**8.61E−05**

*Bold value is *p* < 0.05.*

**TABLE 3:** Working memory transfer.

**Effects**	**AUC**	***B* Estimate**	**Std.**	***p***
		**GINI**	**Err.**	
**Rasch: Random Forest**	0.78	0.55	0.02	**1.05E−97**
Stress–Strain: **Random Forest**	0.69	–0.18	0.03	**2.74E−09**
Diff-Split: **Random Forest**	0.53	–0.49	0.03	**4.68E−49**
**Rasch:** Elastic Net	0.57	–0.41	0.03	**2.65E−37**
Stress–Strain: Elastic Net	0.48	0.00	0.04	9.82E−01
Diff-Split: Elastic Net	0.49	0.33	0.04	**2.05E−14**

*Bold value is *p* < 0.05.*

**TABLE 4:** Working memory cross-validation.

**Effects**	**AUC**	***B* Estimate**	**Std.**	***p***
		**GINI**	**Err.**	
**Rasch: Random Forest: Tomek**	0.92	0.84	0.04	**1.33E−35**
Stress–Strain: **Random Forest: Tomek**	0.91	–0.01	0.05	8.82E−01
Diff-Split: **Random Forest: Tomek**	0.85	–0.14	0.05	**5.89E−03**
**Rasch:** Elastic Net: **Tomek**	0.69	–0.45	0.05	**2.70E−13**
**Rasch: Random Forest:** ENN	0.92	0.01	0.05	8.33E−01
**Rasch: Random Forest:** Unbalanced	0.73	–0.38	0.05	**1.08E−10**
Stress–Strain: Elastic Net: **Tomek**	0.73	0.07	0.07	3.22E−01
Diff-Split: Elastic Net: **Tomek**	0.60	–0.05	0.07	5.22E−01
Stress–Strain: **Random Forest:** ENN	0.92	0.00	0.07	9.89E−01
Diff-Split: **Random Forest:** ENN	0.85	0.00	0.07	9.54E−01
Stress–Strain: **Random Forest:**	0.66	–0.12	0.07	8.68E−02
Unbalanced				
Diff-Split: **Random Forest:** Unbalanced	0.53	–0.26	0.07	**5.72E−04**
**Rasch:** Elastic Net: ENN	0.70	0.00	0.07	9.67E−01
**Rasch:** Elastic Net: Unbalanced	0.51	0.00	0.07	9.81E−01
Stress–Strain: Elastic Net: ENN	0.73	0.01	0.10	9.36E−01
Diff-Split: Elastic Net: ENN	0.60	–0.01	0.10	9.04E−01
Stress–Strain: Elastic Net: Unbalanced	0.63	0.31	0.10	**3.08E−03**
Diff-Split: Elastic Net: Unbalanced	0.53	0.50	0.10	**3.93E−06**

*Bold value is *p* < 0.05.*

**TABLE 5:** Working memory hold-out.

**Effects**	**AUC**	***B* Estimate**	**Std.**	***p***
		**GINI**	**Err.**	
**Rasch: Random Forest**	0.82	0.63	0.07	**2.38E−12**
Stress–Strain: **Random Forest**	0.78	–0.06	0.10	5.06E−01
Diff-Split: **Random Forest**	0.48	–0.68	0.10	**5.94E−09**
**Rasch:** Elastic Net	0.45	–0.74	0.10	**5.57E−10**
Stress–Strain: Elastic Net	0.60	0.38	0.14	**6.89E−03**
Diff-Split: Elastic Net	0.49	0.77	0.14	**7.40E−07**

*Bold value is *p* < 0.05.*

**TABLE 6:** Working memory ISR transfer.

**Effects**	**AUC**	***B* Estimate**	**Std.**	***p***
		**GINI**	**Err.**	
**Rasch: Random Forest**	0.80	0.60	0.02	**1.73E−102**
Stress–Strain: **Random Forest**	0.72	–0.16	0.03	**3.11E−07**
Diff-Split: **Random Forest**	0.50	–0.59	0.03	**4.24E−62**
**Rasch:** Elastic Net	0.49	–0.62	0.03	**5.15E−67**
Stress–Strain: Elastic Net	0.47	0.14	0.04	**2.27E−03**
Diff-Split: Elastic Net	0.50	0.62	0.04	**8.72E−39**

*Bold value is *p* < 0.05.*

#### ISR Model Validation

##### ISR cross-validation

**FIGURE 4:**
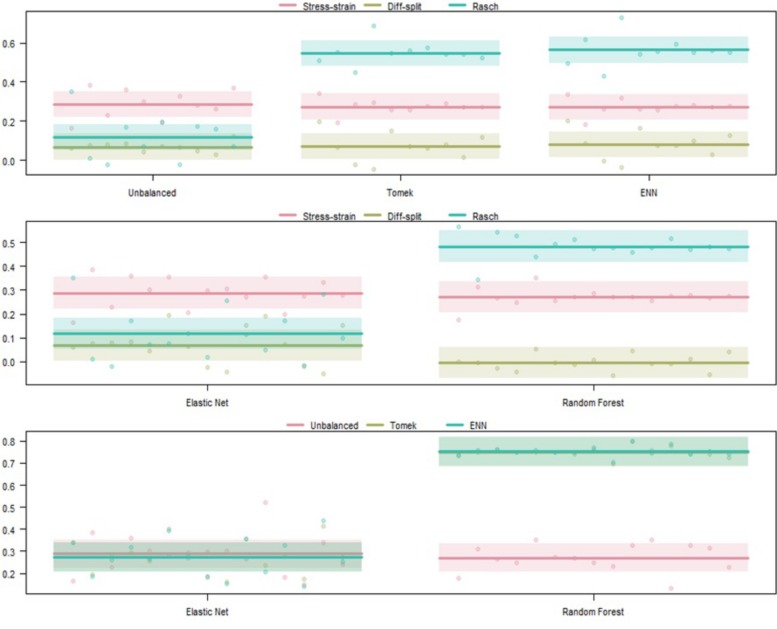
Regression estimates for fivefold cross-validation on the ISR task across various combinations of techniques. Points along the *y*-axis are GINI Index values for individual folds, lines are the regression estimate, and bars are the error of the estimate. **(Top)** Balancing techniques and labeling methods, **(Middle)** machine learning algorithms and labeling methods, and **(Bottom)** machine learning algorithms and balancing techniques.

The results of our regression on the GINI Index values from training cross-validation on the ISR task are presented in Figure 4 and Table 1. The most parsimonious model included a three-way interaction between labeling technique, model, and class balancing technique. First, we see that Rasch labels are significantly better than difficulty split labels. Next, we see that random forests outperformed elastic nets, and that Smote-Tomek and Smote-ENN balancing both outperformed the unbalanced technique. Furthermore both difficulty split and stress–strain labeling techniques, when paired with either elastic nets or an unbalanced dataset, performed significantly worse that Rasch labels with random forests, regardless of dataset balance, or Rasch Labels with Smote-Tomek, regardless of algorithm. Finally, elastic nets paired with unbalanced and either difficulty split or stress–strain labels did not perform as poorly as the main effects and two-way interactions would have predicted. However, this is likely due to it being difficult for the models to perform lower than chance.

##### ISR hold-out participants validation

**FIGURE 5:**
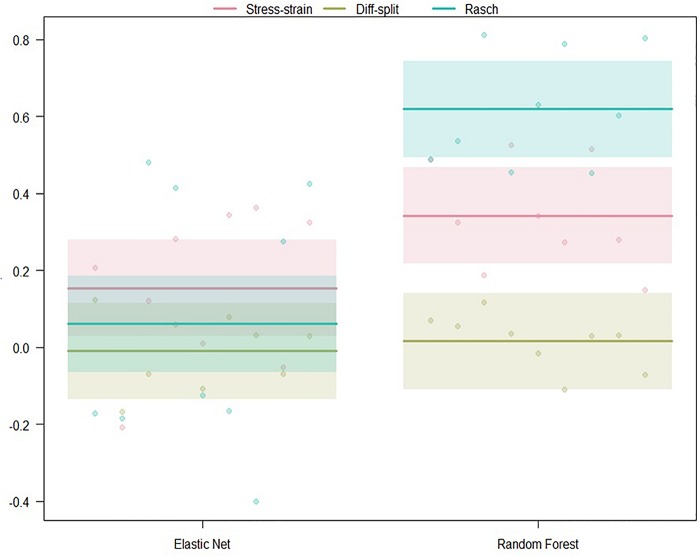
Regression estimates for hold-out participants on the ISR task across labeling technique and machine learning algorithm. Points along the *y*-axis are GINI Index values for individual folds, lines are the regression estimate, and bars are the error of the estimate.

The results of our regression on the GINI Index values from testing our models on the ISR task hold-out participants are presented in Figure 5 and Table 2. The most parsimonious model included a two-way interaction between labeling technique and model. First, we can conclude that Rasch labels are significantly better than stress–strain and difficulty split labels. Next, we see that random forests again outperformed elastic nets. Finally, elastic nets paired with either difficulty split or stress–strain labels did not perform as poorly as the main effects would have predicted. For difficulty split labeling, this is likely because it was difficult for the models to perform lower than chance.

##### ISR transfer to WM validation

**FIGURE 6:**
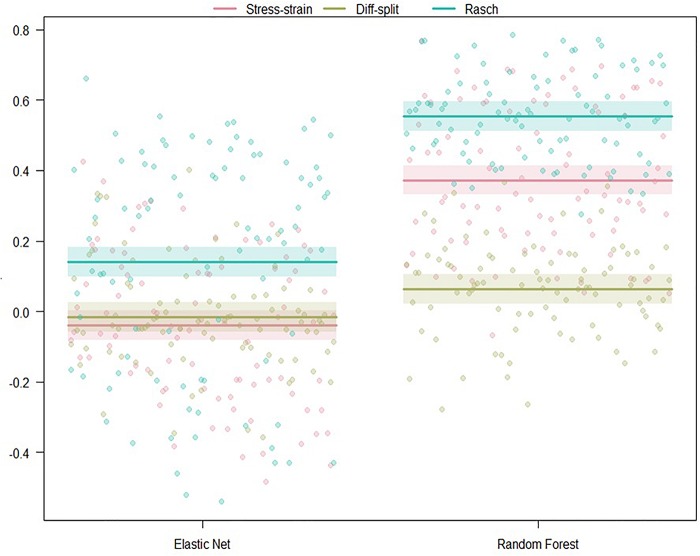
Regression estimates for models trained on the ISR task and transferred to the WM task across labeling technique and machine learning algorithm. Points along the *y*-axis are GINI Index values for individual folds, lines are the regression estimate, and bars are the error of the estimate.

The results of our regression on the GINI Index values from training our models on the ISR task and predicting states in the WM task are presented in Figure 6 and Table 3. The most parsimonious model included a two-way interaction between labeling technique and model. The direction and magnitude of effects are essentially equivalent to what was observed for cross-person transfer in the section *ISR Hold-Out Participants Validation*. However, our error estimates are much lower due to the increased amount of data.

#### WM Model Validation

##### WM cross-validation

**FIGURE 7:**
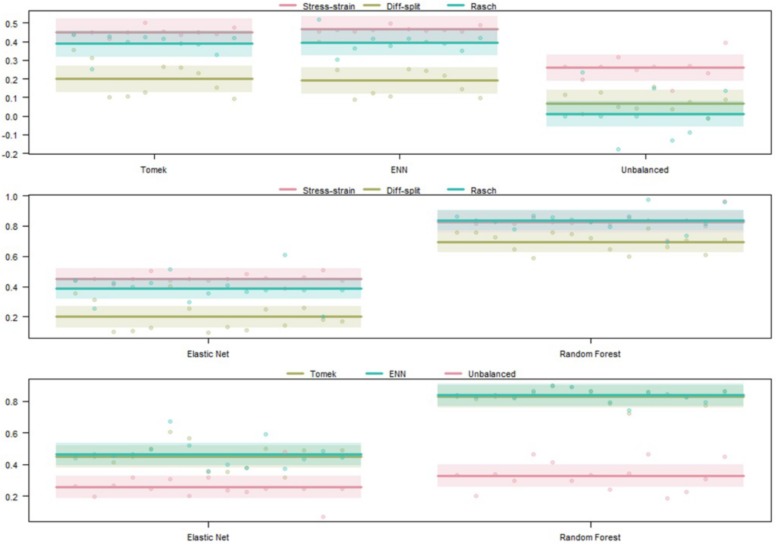
Regression estimates for fivefold cross-validation on the WM task and various combinations of techniques. Points along the *y*-axis are GINI Index values for individual folds, lines are the regression estimate, and bars are the error of the estimate. **(Top)** Balancing techniques and labeling methods, **(Middle)** machine learning algorithms and labeling methods, and **(Bottom)** machine learning algorithms and balancing techniques.

The results of our regression on the cross-validated GINI Index values from training our models on the WM task are presented in Figure 7 and Table 4. The most parsimonious model included a three-way interaction between labeling technique, model, and class balancing technique. First, we see that Rasch labels are significantly better than difficulty split labels. Next, we see that random forests outperformed elastic nets and that Smote-Tomek balancing outperformed the unbalanced technique. Furthermore, difficulty split, when paired with an unbalanced dataset, performed significantly worse than with Smote-Tomek. Finally, elastic nets paired with unbalanced and either stress–strain or difficulty split labels did not perform as poorly as the main effects and two-way interactions would have predicted. However, this is likely because it was difficult for the models to perform lower than chance.

##### WM hold-out participant validation

**FIGURE 8:**
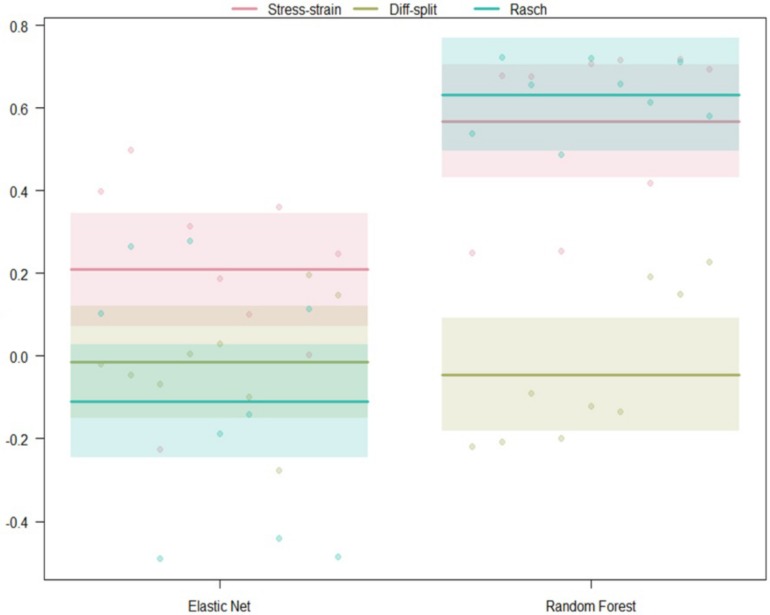
Regression estimates for hold-out participants on the WM task across labeling technique and machine learning algorithm. Points along the *y*-axis are GINI Index values for individual folds, lines are the regression estimate, and bars are the error of the estimate.

The results of our regression on the GINI Index values from testing our models on the WM task hold-out participants are presented in Figure 8 and Table 5. The most parsimonious model included a two-way interaction between labeling technique and model. First, Rasch labels are significantly better than difficulty split labels. Next, we see that random forests again outperformed elastic nets. Finally, elastic nets paired with either stress–strain or difficulty split labels did not perform as poorly as the main effects would have predicted. For difficulty split labeling, this is likely because it was difficult for the models to perform lower than chance.

##### WM transfer to ISR validation

**FIGURE 9:**
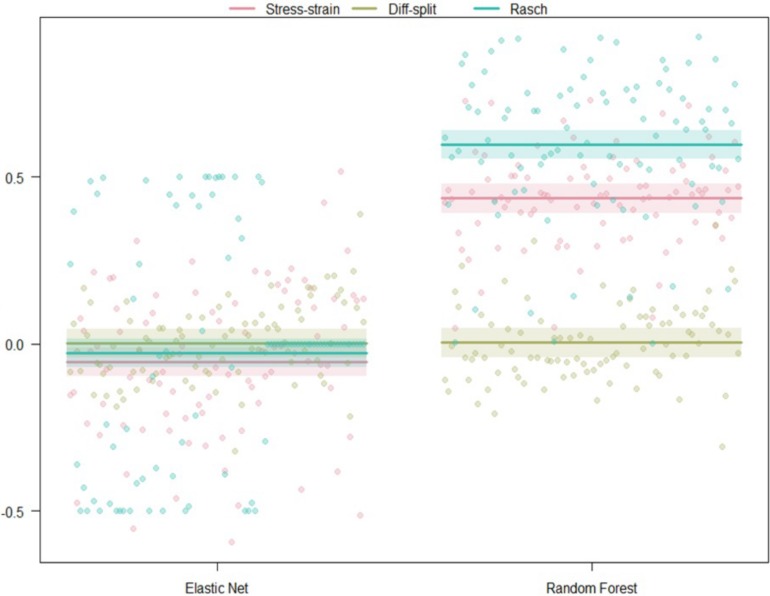
Regression estimates for models trained on the WM task and transferred to the ISR task across labeling technique and machine learning algorithm. Points along the *y*-axis are GINI Index values for individual folds, lines are the regression estimate, and bars are the error of the estimate.

The results of our regression on the GINI Index values from training our models on the ISR task and predicting states in the WM task are presented in Figure 9 and Table 6. The most parsimonious model included a two-way interaction between labeling technique and model. The direction and magnitude of effects are essentially equivalent to what was observed for cross-person transfer in the section *WM Hold-Out Participant Validation*. One addition is that Rasch models also outperformed stress–strain models, and this was likely because error estimates were much lower due to the increased amount of data.

## Discussion

As the presence of automation and advanced intelligent systems become commonplace, there must be methods that can communicate a human’s state of mind to these systems; of particular interest is mental workload. Our work here has accepted mental workload to be a product of the demand/s of the task and the capacity/ies of the person performing the task, where demands and capacities may be moderated by context (Young et al., 2015). In this study, we have tested three methods of labeling mental workload, two algorithms for supervised learning, and three techniques for handling class imbalances. We tested the effects of these methods following, within-task model optimization, hold-out tests with participants not used in the initial training, and cross-task cross-person transfer.

We had clear hypotheses regarding the effects of labeling methods that include individual differences, whereas our tests of algorithms and class imbalance techniques were more exploratory. We tested three hypotheses for the effects of incorporating individual differences into class labeling: (1) this would lead to better initial training and cross-validation of supervised machine learning algorithms, (2) this would lead to better performance of an algorithm trained on person “A” and used to predict the states of person “B,” and (3) this would lead to better performance of an algorithm trained on task “A” and used to predict the states of persons performing task “B.” Notable is the fact that hypothesis 3 assumes that both task “A” and task “B” tax the same cognitive construct, in our case mental workload caused by memory load.

We observed strong evidence that certain algorithms, labeling, and balancing techniques lead to superior training, as measured with cross-validation. The results in the sections *ISR Cross-validation* and *WM Cross-Validation* show clear advantages for random forest algorithms and either of the two Smote data balancing techniques. These differences are consistent across both the ISR and WM tasks. Of greatest interest are the effects observed for the labeling techniques. Across the majority of combinations tested, we see that the labeling techniques that incorporate individual differences (i.e., Rasch and stress–strain) into the state labels lead to better model fits than the alternative. This is in direct support of hypothesis 1. Of note is how poorly models performed on the ISR task when states were labeled solely based on the difficulty of the task (i.e., difficulty split). The use of difficulty for labeling training data was still significantly worse than using a Rasch model even when combined with random forests and Smote-Tomek. The superior model fits from individual difference-based labels provides evidence that mental workload states are moderated by an individual’s traits (i.e., WM capacity).

Our results from the sections *ISR Hold-Out Participants Validation* and *WM Hold-Out Participant Validation* further support the benefits of including individual differences in the labels for supervised training. These sections test hypothesis 2 by testing the effects of transferring states learned on one individual and predicting states in a different individual. Across both the ISR and WM task, we saw that the major effects were coming from the algorithms and how the data were labeled. In support of hypothesis 2, we saw that Rasch labeling produced the best-fitting models. Furthermore, the trends of performance for the algorithms and labeling methods were similar across the ISR and WM tasks. Most importantly, we observed that labels based solely on task difficulty performed no better than chance. Yet, both labeling techniques that used individual differences when paired with random forests produced better than chance results. Again, the Rasch model-based labels provided quite good model fits across all participants not in the initial optimization and training steps. The worst-fitting random forest Rasch model on an individual across both tasks was 0.45 on the GINI index; this was as good as the best individual fit when elastic nets were employed in the same context.

The support for including individual differences in supervised learning labels is further bolstered by our observations in the sections *ISR Transfer to WM Validation* and *WM Transfer to ISR Validation*. In these sections, we test hypothesis 3, regarding how well a model would perform when trained on a group of individuals in one task and then applied to a different group of individuals on a different task. Again, we saw that the majority of variance in model fit could be explained by the algorithms and labeling techniques. We, also again, observed the best model fits with Rasch labeling. This trend was consistent regardless of whether the model was trained on the ISR or WM task. These model fits were also similar to what was observed in the previous sections. Specifically, random forests paired with either of the individual difference label techniques were the only models that consistently learned, providing clear support for hypothesis 3.

Overall, our results provide strong support for our hypothesis that to robustly predict mental states, individual differences should be encoded into the labels used by supervised learning algorithms. We see that when individual differences are included, we can produce good model fits during initial training but most importantly also during transfer.

Consistent transfer of mental workload state prediction across individuals and across tasks is a first step toward broad use of generalizable passive brain–computer interfaces (Zander and Kothe, 2011). In fNIRS, EEG, and fMRI, researchers have shown person generalizable models to be possible (Mitchell et al., 2004; Grimes et al., 2008; Wang et al., 2011; Yuksel et al., 2016). Furthermore, within these modalities, there is evidence that cognitive state models trained on one task can correlate with or enhance performance on a separate task (Berka et al., 2007; Yuksel et al., 2016). Supervised models have also been previously shown to transfer across different kinds of stimuli within a common task structure (Grimes et al., 2008). Our work extends these accomplishments in two ways. First, we show cross-person and cross-task transfer simultaneously, across both groups and tasks. It is also important to note that this occurs in two tasks that have different stimuli, encoding, and response schemas. Second, we demonstrate that these models don’t just correlate with or enhance task performance but can predict specific mental states in a different task.

The success of both individual difference labeling techniques, and Rasch labeling in particular, provides evidence that it may be possible to develop individual and task independent classifiers of specific mental workload states. It may be possible that additional classifiers could be created for cognitive states beyond memory. For example, a classifier that reflects the transition points between early and late attentional filtering as described in perceptual load theory (Lavie et al., 2004). Perhaps even more ambitious, many classifiers could be run in parallel accounting for different cognitive resource pools. Following Multiple Resource Theory software systems could adapt how humans interact within machine systems to take advantage of the channels with the highest bandwidth. There has already been success in adapting learning *via* passive brain–computer interfaces, yielding improved performance, engagement, and enjoyment in the learning task (Yuksel et al., 2016).

### Limitations and Future Work

We see this work as a starting point to understanding the benefits of bolstering supervised learners with individual differences information; in that regard, the study was not without limitations. First, we only compared two supervised learning algorithms. It is possible that other algorithms can produce superior performance to random forests and this should be explored. It may be possible that simpler algorithms can produce similar results to those observed with random forests. In this sense, this is why we are not prepared to say that regularized algorithms are poor performers on this type of data. In our case, many of our features were polynomial, which led to high correlations between our features; elastic nets do not handle this well. There may be other regularized algorithms that deal better with correlated features; we plan to explore this in the future. Transitioning to the tasks we used, both of which were externally paced and relatively basic, and because they were examined under controlled conditions, only one parameter (memory load) was allowed to vary. To further vet mental state prediction, tests need to be performed on more applied, self-paced tasks where the solutions and responses are more open-ended. Further, we plan to explore the robustness of mental state predictors in the context of more varied environments where multiple cognitive parameters are allowed to vary, and separate states may be elicited for them.

## Conclusion

The aim of the current study was to assess the importance of incorporating individual differences into the labeling schema for supervised machine learning to predict mental workload states from neurophysiological data. Within this context, we also wanted to explore the effects of two different algorithms for supervised learning (i.e., random forests and elastic nets) as well as the effects of different methods for handling class imbalances for supervised learning [i.e., Smote-Tomek, Smote-ENN, and modifying class weights (unbalanced)]. To assess these effects, we tested the model fits of each label, algorithm, and balancing combination during cross-validation, cross-person transfer, and cross-task transfer. We observed strong model fits across all test conditions only when using a combination of labels incorporating individual differences and random forests learners. We take this as evidence that supervised learners used for neurophysiological mental state prediction greatly benefit from labels that incorporate individual differences. Furthermore, these findings provide evidence that it may be possible to develop person- and task-independent classifiers of specific mental states, when states are defined *via* individual differences modeling.

## Data Availability

The datasets generated for this study are not publicly available because of Northrop Grumman Corporation’s data sharing policies. Requests to access the datasets should be directed to the corresponding author.

## Author Contributions

BFa, BFe, AH, and RM designed the tasks. BFe programmed the tasks. BFa and AH completed the data collection. BFe and RM completed the data analysis. BFa, BFe, AH, and RM prepared the final manuscript.

## Conflict of Interest Statement

RM, BFe, AH, and BFa were employed by Northrop Grumman. The authors declare that this study received funding from Northrop Grumman. No Northrop Grumman employee other than the aforementioned authors was involved in the study design, data collection, analysis, and interpretation of study results presented in this manuscript.

## Footnotes

1 www.fnirdevices.com
